# Identification and comparison of heart-rate dynamics during cycle ergometer and treadmill exercise

**DOI:** 10.1371/journal.pone.0220826

**Published:** 2019-08-22

**Authors:** Kenneth J. Hunt, Reto Grunder, Andreas Zahnd

**Affiliations:** Institute for Rehabilitation and Performance Technology, Division of Mechanical Engineering, Department of Engineering and Information Technology, Bern University of Applied Sciences, Burgdorf, Switzerland; University of Bourgogne France Comté, FRANCE

## Abstract

**Aim and methods:**

The aim of this study was to compare the dynamics of heart rate (HR) response to exercise using a cycle ergometer (CE) and a treadmill (TM). Using a sample of 25 healthy male participants, the time constant of HR dynamics was estimated for both modalities in response to square-wave excitation.

**Results:**

The principal finding was that the time constant of heart-rate dynamics around somewhat-hard exercise intensity (Borg rating of perceived exertion = 13) does not differ significantly between the CE and TM (68.7 s ± 21.5 s vs. 62.5 s ± 18.5 s [mean ± standard deviation]; CE vs. TM; *p* = 0.20). An observed moderate level of evidence that root-mean-square model error was higher for the CE than for the TM (2.5 bpm ± 0.5 bpm vs. 2.2 bpm ± 0.5 bpm, *p* = 0.059) may reflect a decrease in heart rate variability with increasing HR intensity because, in order to achieve similar levels of perceived intensity, mean heart rate for the CE was ∼25 bpm lower than for the TM.

**Conclusion and significance:**

These results have important implications for model-based design of automatic HR controllers, because, in principle, the same dynamic controller, merely scaled according to the differing steady-state gains, should be able to be applied to the CE and TM exercise modalities.

## 1 Introduction

Exercise intensity is a key element in the prescription of training programmes. Contemporary guidelines for development and maintenance of fitness recommend specific ranges for weekly duration and frequency of training sessions [1, 2], while different intensity levels for individual exercise bouts can be flexibly combined, such as in high-intensity interval training (HIIT) [3, 4]. Intensity regimes can be described in relation to age-predicted or actual maximal heart rate (HRmax) or heart rate reserve (HRR) [1, 2], or using a rating of perceived exertion (RPE) scale [5, 6].

Many approaches to modelling of HR dynamic are available [7, 8], including HR response to gross changes in exercise intensity during cycling [9]. In exercise physiology, it is common to consider three-phase models comprising the rapid cardiodynamic response (Phase I), the major but slower Phase II increase, and, provided the intensity is above the anaerobic threshold, a small but prolonged Phase III component [10, 11].

In contrast, when a model of HR dynamics is required for feedback control synthesis, the prevalent approach is to linearise the system by considering small-signal deviations around a nominal input-output operating point [12, 13]. Such models have been used for model-based design of feedback controllers that automatically adjust the manipulated variable (e.g. treadmill speed) to maintain actual HR close to a target profile [14], whereby the main challenge is to deal appropriately with disturbances caused by broad-spectrum heart rate variability (HRV) [15].

It is therefore of relevance to investigate control-orientated dynamic models of HR using the two most prevalent exercise modalities, namely cycle ergometers and treadmills: the aim of this study was to compare the dynamics of heart rate response to exercise using a cycle ergometer (CE) and a treadmill (TM).

## 2 Materials and methods

### 2.1 Ethics and participants

Ethical approval for this study was granted by the Ethics Committee of the Swiss Canton of Bern (Ref. 2017-01894). A convenience sample of 25 males was drawn that included participants aged between 18 and 35 years who were regular exercisers (at least three exercise sessions per week, each of duration at least 30 min), non-smokers, had no prior history of cardiovascular or respiratory disease, and had no current musculoskeletal complaints or injuries. Convenience sampling was conducted by the authors by personal approach to members of the undergraduate cohort in their institution. All participants gave written, informed consent in accordance with the Declaration of Helsinki.

Demographics of the sample were (mean ± standard deviation [range]): age/(years) = 25.2 ± 2.6 (22 to 32); mass/(kg) = 79.4 ± 12.2 (62 to 114); height/(m) = 1.82 ± 0.07 (1.65 to 1.93); body mass index/(kg/m^2^) = 24.0 ± 3.5 (19.9 to 34.0).

### 2.2 Test procedures

The study had a repeated-measures crossover design where each participant performed one test on each exercise device. Counterbalancing was employed to eliminate any effect of the order of presentation, i.e. TM then CE vs. CE then TM: presentation order was sequentially changed and participants were randomly assigned upon recruitment. Participants were required to avoid strenuous activity within the 24 hours prior to each test, to refrain from caffeine for 12 hours before, and not to consume a large meal within 3 hours prior to testing. There was at least 48 hours between tests.

**Fig 1:**
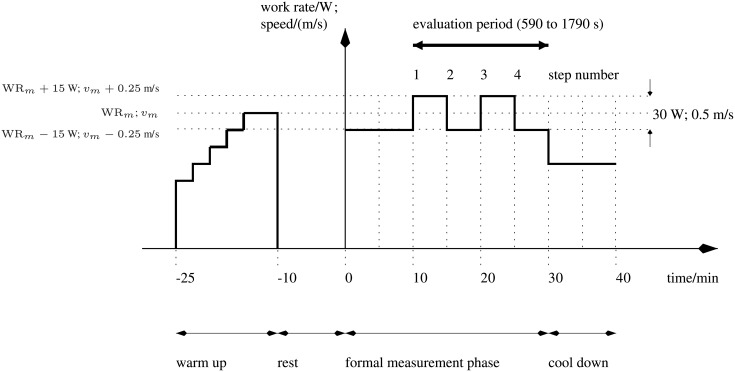
Test protocol for the cycle ergometer (work rate/(W)) and treadmill (speed/(m/s)).

During the formal measurement phase of each test (Fig 1), the manipulated variable (CE—work rate, WR; TM—speed, *v*;) was implemented as a square wave. Square-wave excitation is known from system identification theory to be sufficiently exciting for the simple, first-order model structure employed here (Eq (1)), whereas, for higher-order models, more complex inputs such as pseudo-random binary sequence (PRBS) signals would be required [16].

The mid-level of the square wave was adjusted individually to correspond approximately to the boundary between moderate and vigorous exercise intensities. In terms of the Borg RPE scale [5, 6], this boundary has the value 13 (“somewhat hard”). When using HR, the boundary value is dependent on the exercise modality, because of the known differences in perceived intensity of treadmill and cycle exercise [17]. For the TM, the boundary was defined as HR* = 0.765 x HRmax = 0.765 x (220—age) [2, 18]. For the CE, the target mean HR* was set 20 bpm lower on the basis of data demonstrating that heart rate at the “individual anaerobic threshold” (IAT) is approximately 20 bpm lower during cycling [19]: the goal in the present study was to achieve similar perceived exercise intensities close to the moderate-vigorous boundary, i.e. corresponding roughly to the IAT, for both modalities. For the CE, participants were instructed to maintain a constant cycling cadence of 70 rpm; to do this, they observed a digital cadence display mounted on the handlebars.

Each test comprised four stages (Fig 1):

1. Warm up (15 min [900 s]): the manipulated variable was automatically adjusted using an existing feedback controller to achieve the target HR* as defined above. The mean value of the manipulated variable during the period 650 s to 850 s was taken as the mid-level value for the formal measurement phase (TM—*v*_*m*_; CE—WR_*m*_).
2. Rest (10 min).
3. Formal measurement phase (30 min): the manipulated variable was changed in the form of a square-wave signal for 30 min with variations around the mid-level as calculated above. For the TM, the amplitude of the signal was 0.25 m/s (i.e. *v* = *v*_*m*_ ± 0.25 m/s) and for the CE it was 15 W (i.e. WR = WR_*m*_ ± 15 W).
4. Cool down (10 min): the manipulated variable was individually set to a low level.

### 2.3 Equipment and data collection

The treadmill (model Venus, h/p/cosmos Sports & Medical GmbH, Germany) and cycle ergometer (model LC7, Monark Exercise AB, Sweden) were connected via serial link to a PC and controlled in real time using a Matlab/Simulink model (The Mathworks, Inc., USA). Heart rate was monitored using a chest belt (model T34, Polar Electro Oy, Finland) and transmitted wirelessly to a receiver module (Heart Rate Monitor Interface [HRMI], Sparkfun Electronics, USA) connected via serial cable to the PC and the Simulink model. HR data were recorded with a sample period of 1 s and downsampled to 5 s for model identification by averaging the current and four preceding values at each 5 s sample point.

The individually-perceived intensity of exercise was manually recorded during the formal measurement phase using the Borg RPE scale. RPE was taken one minute before the end of each of the four five-minute-duration step changes in the manipulated variable (Fig 1), i.e. at times 14 min (840 s), 19 min (1140 s), 24 min (1440 s) and 29 min (1740 s); these four values were averaged for each individual test.

### 2.4 Outcome measures and statistical analysis

The responses of HR to changes in the manipulated variable (TM—speed, *v*; CE—work rate, WR) were modelled as first-order linear time-invariant transfer functions,

$$\begin{array}{c} v,\text{WR}\to \text{HR}:\;P_{o}\left(s\right)=\frac{k}{\tau s+1}, \end{array}$$ (1)

with time constant *τ* and steady-state gain *k*.

For each individual identification test, estimates of *τ* and *k* in Eq (1) were obtained by least-squares optimisation using the Matlab System Identification Toolbox (The Mathworks, Inc., USA). Goodness-of-fit was quantified using the absolute root-mean-square error (RMSE) between the model-simulated and measured outputs. These values were calculated over an evaluation period from 590 to 1790 s of the formal measurement phase (Fig 1). Prior to parameter estimation, input-output data were detrended and mean levels were subtracted. Mean values of *τ* were compared to test for any differences in HR dynamics between the CE and TM, and RMSE was also compared. *k* cannot be compared between the devices as the units are not the same (bpm/W vs. bpm/(m/s)).

To evaluate the efficacy of the method of setting the perceived intensity of exercise for the two modalities, where target HR was set 20 bpm lower for the CE, average RPE and mean HR were compared over the evaluation period.

For hypothesis testing, normality of the sample differences was checked using the Kolmogorov-Smirnov test with Lilliefors correction. For normal data, paired two-sided t-tests were employed; Wilcoxon signed rank tests were used otherwise; the significance level was *α* = 0.05. The Matlab Statistics and Machine Learning Toolbox (The Mathworks, Inc., USA) and R (R Foundation for Statistical Computing, Austria) were used.

## 3 Results

**Fig 2:**
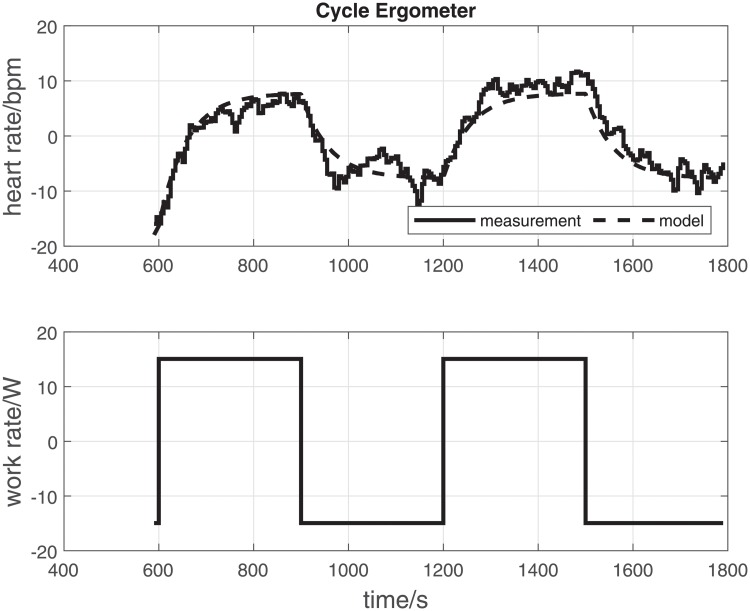
CE: Original data records and parameter estimation results for a single participant (participant No. 14) on the cycle ergometer. The upper panel shows the measured and model-simulated heart rate. The lower panel shows the manipulated variable (CE—work rate). Input-output data were detrended and mean levels subtracted prior to estimation. *τ* = 57.3 s, *k* = 0.51 bpm/W, RMSE = 2.3 bpm.

**Fig 3:**
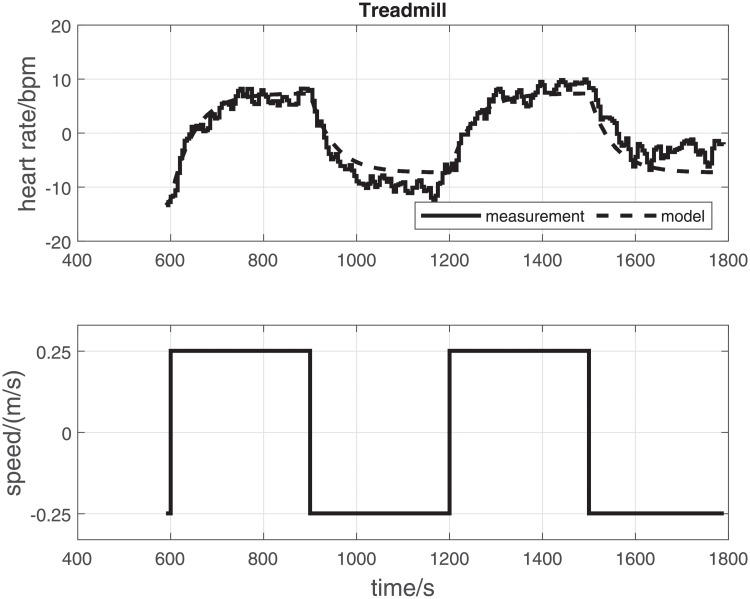
TM: Original data records and parameter estimation results for a single participant (participant No. 14) on the treadmill. The upper panel shows the measured and model-simulated heart rate. The lower panel shows the manipulated variable (TM—speed). Input-output data were detrended and mean levels subtracted prior to estimation. *τ* = 49.7 s, *k* = 29.4 bpm/(m/s), RMSE = 2.4 bpm.

To illustrate the data processing method and outcome measures, representative original data records for one participant are provided (CE—Fig 2; TM—Fig 3).

**Fig 4:**
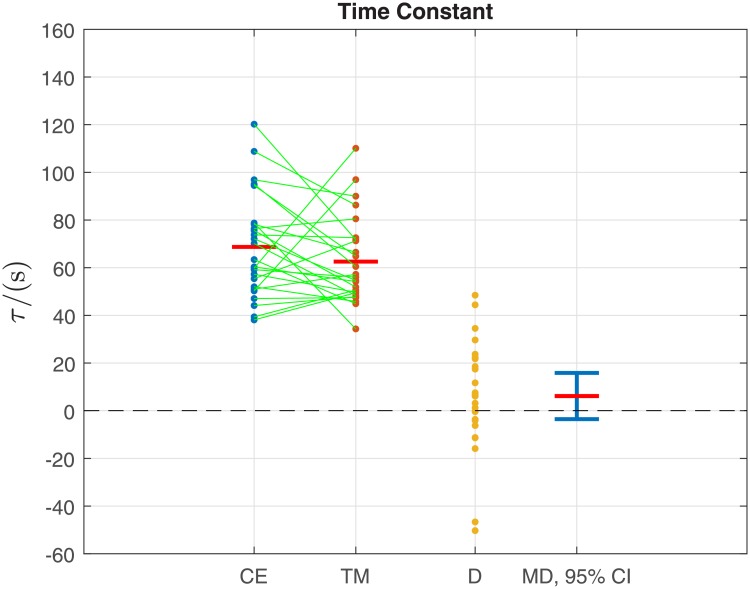
Primary outcome: Data samples for time constant *τ* for all 25 participants for the cycle ergometer CE and treadmill TM (see also Table 1). The green lines link the sample pairs from each participant. The red horizontal bars depict mean values (given numerically in Table 1). D = CE—TM is the difference between the paired samples. MD is the mean difference (red horizontal bar), with its 95% confidence interval (CI) in blue. The value 0 is within the 95% CI, indicating no significant difference between the means: this conforms with *p* > 0.05 for this variable (Table 1).

Overall, the mean time constant *τ* for the cycle ergometer and treadmill did not differ significantly (68.7 s ± 21.5 s vs. 62.5 s ± 18.5 s [mean ± standard deviation]; CE vs. TM; *p* = 0.20; Table 1; Fig 4). Similarly, mean RMSE for the estimated CE and TM models was not significantly different (2.5 bpm ± 0.5 bpm vs. 2.2 bpm ± 0.5 bpm; *p* = 0.059; Table 1).

**Table 1:** Outcomes for cycle ergometer vs. treadmill and *p*-values for comparison of means (25 participants).

	mean ± SD (range)		MD (95% CI)	*p*-value
	CE	TM	CE—TM	
*τ*/s	68.7 ± 21.5 (38.1, 120.2)	62.5 ± 18.5 (34.3, 110.1)	6.2 (-3.5, 15.9)	0.20
RMSE/bpm	2.5 ± 0.5 (1.3, 3.6)	2.2 ± 0.5 (1.3, 3.1)	0.3 (-0.01, 0.6)	0.059
RPE/(6–20)	12.6 ± 1.4 (10.2, 15.8)	12.8 ± 1.4 (10.0, 15.8)	-0.4 (-0.7, 0.2)	0.24
mean HR/bpm	129.9 ± 3.8 (125.0, 139.6)	154.7 ± 4.9 (139.1, 164.9)	-24.9 (-27.1, -22.7)	5.2 × 10^−18^
*k*/(CE: bpm/W; TM: bpm/(m/s))	0.392 ± 0.120 (0.180, 0.796)	26.2 ± 11.1 (13.3, 62.9)	na	na

CE: cycle ergometer

TM: treadmill

SD: standard deviation

MD: mean (*τ*, RMSE, mean HR) or median (RPE) difference of CE—TM

95% CI: confidence interval for the mean (*τ*, RMSE, mean HR) or median (RPE) difference

*p*-values: paired two-sided t-tests (*τ*, RMSE, mean HR) or Wilcoxon signed-rank test (RPE)

*τ*: time constant

RMSE: root-mean-square error

RPE: rating of perceived exertion (Borg scale)

HR: heart rate

*k*: steady-state gain

na: not applicable

bpm: beats per minute

On average, the perceived exercise intensity RPE was close to the target value of 13 (“somewhat hard”) for both the CE (12.6 ± 1.4) and the TM (12.8 ± 1.4), and the difference between the two modalities was not significant (*p* = 0.23, Table 1). Concomitantly, mean heart rate for the CE was approximately 25 bpm lower than for the TM (129.9 bpm ± 3.8 bpm vs. 154.7 bpm ± 4.9 bpm; *p* = 5.2 × 10^−18^; Table 1).

For the CE, actual mean HR was very close to the age-predicted target mean HR (129.9 bpm ± 3.8 bpm vs. 128.7 bpm ± 2.0 bpm; 1.1 (-0.4, 2.7) [mean difference (95% confidence interval)]; *p* = 0.15). For the TM, actual mean HR was 6 bpm higher than the target value (154.7 bpm ± 4.9 bpm vs. 148.7 bpm ± 2.0 bpm; 6.0 (4.1, 7.9); *p* = 1.2 × 10^−6^).

For the treadmill, the mean steady-state gain *k* and time constant *τ* in the present study was compared to the values obtained in a previous TM study in a separate cohort of 25 participants [12]: neither *k* (mean values 26.2 bpm/(m/s) vs. 24.2 bpm/(m/s); *p* = 0.39) nor *τ* (62.5 s vs. 57.6 s; *p* = 0.36) were significantly different between the studies (present vs. previous studies; independent-samples t-tests).

## 4 Discussion

The principal finding of this study was that heart-rate dynamics around somewhat-hard exercise intensity (RPE = 13) do not differ significantly between the cycle ergometer and treadmill.

There was a moderate level of evidence that RMS model error was higher for the CE than for the TM (RMSE 2.5 bpm vs. 2.2 bpm, CE vs. TM, *p* = 0.059; Table 1). Since the RMSE function is closely related to the time-domain heart rate variability (HRV) measure SDNN (standard deviation of all normal-to-normal intervals, [20, 21]), this result may reflect the observation that HRV decreases with increasing HR intensity [22, 15], because mean HR for the CE was ∼25 bpm lower than for the TM (Table 1).

The methodology employed here to match the perceived exercise intensities for the CE and TM, namely the setting of target mean HR to 20 bpm lower for the CE, can be considered to have been highly successful because mean RPE for the CE and TM were very similar and very close to the target value of 13 (12.6 vs. 12.8, *p* = 0.23, Table 1). It is also of note that, for the CE, the actual mean HR of 129.9 bpm is very close to the HR that nominally corresponds to an RPE of 13, i.e. 130 bpm: by design, the Borg RPE scale linearly increases by a factor of 10 in relation to HR for cycle ergometer exercise [23].

The findings of the present study, that HR dynamics do not differ between the modalities, and that HRV appears to decrease with increasing HR intensity, have important practical implications, particularly in relation to the model-based design of automatic HR controllers. With regard to HRV, it has previously been observed that the principal challenge in HR-control design is to deal appropriately with disturbances caused by broad-spectrum HRV [14]; furthermore, in concordance with the results obtained here, HRV was demonstrated to decrease with exercise intensity [15]. This implies, conversely, that achieving accurate control of HR will be more difficult at lower intensities.

With regard to HR dynamics, the observation of similar time constants raises the possibility that the same dynamic controller, merely scaled according to the differing steady-state gains, might be applied to both the CE and TM. In a follow-on study [24], this hypothesis was found to hold when a single HR controller was used for both the CE and TM in the same participant cohort used in the present study: using a model with time constant taken as the overall mean of the CE and TM values obtained here (i.e. *τ* = 65.6 s, Table 1), mean RMS HR tracking errors for the CE and TM were very low and not significantly different (3.1 bpm vs. 2.8 bpm, CE vs. TM, *p* = 0.13).

## Supporting information

S1 File S1_File.zip: Cycle ergometer data files. The input (work rate) is the variable “Signal_Power_ident”; the output (heart rate) is the variable “BPM_HRMI”.
(ZIP)

S2 File S2_File.zip: Treadmill data files. The input (speed) is the variable “speed_XX”; the output (heart rate) is the variable “HR_ist_XX” where XX refers to the file number.
(ZIP)

## Nomenclature

bpm: beats per minute
CE: cycle ergometer
CI: confidence interval
HIIT: high intensity interval training
HR: heart rate
HR*: target heart rate
HRmax: maximal heart rate
HRR: heart rate reserve
HRV: heart rate variability
*k*: steady-state gain
MD: mean difference
NN: normal-to-normal
RMS: root-mean-square
RMSE: root-mean-square error
RPE: rating of perceived exertion
rpm: revolutions per minute
SD: standard deviation
SDNN: standard deviation of all normal-to-normal intervals
TM: treadmill
*v*: speed
*v*_*m*_: mid-level speed
WR: work rate
WR_*m*_: mid-level work rate
*τ*: time constant
